# Prognostic impact of chromosomal aberrations in Ewing tumours

**DOI:** 10.1038/sj.bjc.6600332

**Published:** 2002-06-05

**Authors:** C M Hattinger, U Pötschger, M Tarkkanen, J Squire, M Zielenska, S Kiuru-Kuhlefelt, L Kager, P Thorner, S Knuutila, F K Niggli, P F Ambros, H Gadner, D R Betts

**Affiliations:** CCRI, St. Anna Children's Hospital, A-1090 Vienna, Austria; Department of Medical Genetics, Haartman Institute, University of Helsinki, FIN-00014 Helsinki, Finland; Laboratory of Medical Genetics, Helsinki University Central Hospital, FIN-00014 Helsinki, Finland; Ontario Cancer Institute and Medical Biophysics, Faculty of Medicine, University of Toronto, Toronto, Ontario M5G 2M9, Canada; Department of Laboratory Medicine and Pathobiology, Faculty of Medicine, University of Toronto, Toronto, Ontario M5G 2M9, Canada; Division of Pathology, Department of Pediatric Laboratory Medicine, Hospital for Sick Children, Toronto, Ontario M5G 2M9, Canada; St. Anna Children's Hospital, A-1090 Vienna, Austria; Department of Oncology, University Children's Hospital, CH-8032 Zürich, Switzerland

**Keywords:** Ewing tumours, prognostic markers, molecular cytogenetics, 1q, 16q, chromosome 12

## Abstract

Although greater than 50% of Ewing tumours contain non-random cytogenetic aberrations in addition to the pathognomonic 22q12 rearrangements, little is known about their prognostic significance. To address this question, tumour samples from 134 Ewing tumour patients were analysed using a combination of classical cytogenetics, comparative genomic and fluorescence *in situ* hybridisation. The evaluation of the compiled data revealed that gain of chromosome 8 occurred in 52% of Ewing tumours but was not a predictive factor for outcome. Gain of 1q was associated with adverse overall survival and event-free survival in all patients, irrespective of whether the tumour was localised or disseminated (overall survival: *P*=0.002 and *P*=0.029; event-free survival: *P*=0.018 and *P*=0.010). Loss of 16q was a significant predictive factor for adverse overall survival in all patients (*P*=0.008) and was associated with disseminated disease at diagnosis (*P*=0.039). Gain of chromosome 12 was associated with adverse event-free survival (*P*=0.009) in patients with localised disease. These results indicate that in addition to a 22q12 rearrangement confirmation in Ewing tumours it is important to assess the copy number of 1q and 16q to identify patients with a higher probability of adverse outcome.

*British Journal of Cancer* (2002) **86**, 1763–1769. doi:10.1038/sj.bjc.6600332 www.bjcancer.com

© 2002 Cancer Research UK

## 

Skeletal and extraskeletal Ewing's sarcoma (ES) and peripheral primitive neuroectodermal tumours (pPNET) are characterised by a high expression of the CD99 antigen (Ambros *et al*, 1991) and the presence of the balanced translocation t(11;22)(q24;q12) or related aberrations involving 22q12 (Aurias *et al*, 1983; Turc-Carel *et al*, 1983; Becroft *et al*, 1984; Whang-Peng *et al*, 1984). Therefore, small-blue-round-cell tumours of childhood and early adolescence that show high CD99 expression and the presence of a 22q12 aberration are often grouped under the term ‘Ewing tumours’ (ETs) (Ambros *et al*, 1991) or ‘Ewing family of tumours’ (EFTs) (Delattre *et al*, 1994).

The t(11;22)(q24;q12) that is present in 85–90% of ETs (Mitelman *et al*, 2001) generates a chimeric fusion transcript between the *EWS* (22q12) and *FLI1* (11q24) genes (Delattre *et al*, 1992; Zucman *et al*, 1992). In the remaining cases, the *EWS* gene is rearranged with other partners of the *ETS* oncogene family (for review see Sandberg and Bridge, 2000). In addition to the rearrangements involving 22q12, non-random chromosomal aberrations occur in more than 50% of cytogenetically analysed ETs (Sandberg and Bridge, 2000; Mitelman *et al*, 2001). Chromosome gain is the most frequent event, of which trisomy 8 is the most common found in almost 50% of the cases, with gains of 2, 12 and 20 also being frequent non-random events (Mugneret *et al*, 1988; Kullendorff *et al*, 1999). Additional structural aberrations are less common than numerical changes, although unbalanced rearrangements involving chromosomes 1 and 16 are quite frequently seen. In the majority of these cases the net imbalance is gain of 1q with simultaneous loss of 16q (Mugneret *et al*, 1988; Douglass *et al*, 1990; Hattinger *et al*, 1996; Armengol *et al*, 1997; Stark *et al*, 1997; Kullendorff *et al*, 1999; Tarkkanen *et al*, 1999).

To date only the presence of metastases at diagnosis has constantly been reported to be a negative prognostic marker for ET patients (Jürgens *et al*, 1988; Terrier *et al*, 1996; Cotterill *et al*, 2000). In addition, in patients with localised disease, poor histological response to chemotherapy (Picci *et al*, 1997; Bacci *et al*, 2000), tumour volume (Jürgens *et al*, 1988; Koscielniak *et al*, 1992; Ahrens *et al*, 1999), primary tumour site and age less than 15 years at diagnosis have been variably associated with adverse clinical outcome (Cotterill *et al*, 2000).

Clinical implications of genetic changes in ETs are poorly understood. Type 1 *EWS*-*FLI1* fusion transcripts were associated with better outcome in patients with localised disease compared to all other *EWS* fusion transcripts in few studies (Zoubek *et al*, 1994, 1996; de Alava *et al*, 1998). Recently, deletions of *INK4A* (9p21) and *TP53* alterations (17p13) appeared to define small groups of patients with markedly poor outcome (Kovar *et al*, 1997; de Alava *et al*, 2000; Wei *et al*, 2000). Besides molecular genetic markers of possible prognostic value, increased copy number of chromosomes 8, 12, and of 1q and loss of 1p have been discussed to be associated with an advanced stage of disease, but with conflicting evidence as to whether they are associated with a poor clinical outcome (Douglass *et al*, 1990; Armengol *et al*, 1997; Maurici *et al*, 1998; Hattinger *et al*, 1999; Kullendorff *et al*, 1999; Tarkkanen *et al*, 1999). As ETs are rare all these previous studies were performed on small numbers of patients and could not reach firm conclusions with regard to the prognostic impact of the additional genetic aberrations.

To ascertain the clinical significance of the most frequent additional cytogenetic events in a large series of patients with ET, the present collaborative retrospective study was initiated. Genetic data from clinically well documented ET patients were collected and statistical analyses were performed in order to elucidate correlations between genetic and clinical parameters and to determine the influence of these parameters on the clinical outcome.

## PATIENTS AND METHODS

### Patients and tumour specimens

Genetic and clinical data were collected from a series of 146 patients with ET from four different centres (Helsinki, Toronto, Vienna, Zurich). Criteria for inclusion in the study were date of diagnosis between January 1983 and October 1999 and confirmed diagnosis of ET either by positive CD99 staining and/or the presence of a 22q12 rearrangement. Due to incomplete clinical and/or genetic data 12 patients were excluded from statistical analyses. Thus, 134 patients were included in the final study representing consecutive series of ET patients for each of the four centres: Helsinki (23 patients), Toronto (24 patients), Vienna (68 patients) and Zurich (19 patients).

The median age at diagnosis of ET was 13 years (range 4 months–37 years). Sixty-two patients were male (46%) and 72 female (54%). Chest wall (*n*=15), pelvis (*n*=14) and femur (*n*=12) were the most frequent tumour sites at diagnosis in the 91 patients presenting with localised ET. The primary tumours of the 43 patients with disseminated disease at diagnosis were located most frequently in the pelvis (*n*=15). Other tumour locations were found in less than 10 patients each. In 121 patients, tumour tissue at diagnosis was analysed, in four patients at both diagnosis and relapse, and in nine patients at relapse. Published chemotherapy regimens were applied to 120 patients: CESS-81 and CESS-86+91P (Jürgens *et al*, 1988) in two patients each, CESS-86 (Jürgens *et al*, 1988) in 18 patients, CESS-91P (Jürgens, 1994) and Ewing-SF (Miser *et al*, 1987) in four patients each, EICESS-92 (Zoubek *et al*, 1996) in 40 patients, CWS (Koscielniak *et al*, 1992) in five patients, SSG IX (Elomaa *et al*, 2000) in 16 patients, SSG IX with reduced doses in one patient, Ewing-CA (Zielenska *et al*, 2001) in 24 patients and Ewing-IL (Stark *et al*, 1997) in three patients. One patient received palliative therapy (Wiklund *et al*, 1992) after first relapse only. Thirteen patients received chemotherapy regimens similar to published protocols used in the other centres, and one patient did not receive chemotherapy.

### Genetic analysis

Tumour samples were analysed with complementary techniques. Chromosome analysis was performed in all four centres according to standard protocols (Dracopoli, 2001; ISCN, 1995). Cytogenetic data were available in 93 cases, of which 68 have been published in detail (Hattinger *et al*, 1996, 1999; Armengol *et al*, 1997; Stark *et al*, 1997; Tarkkanen *et al*, 1999; Zielenska *et al*, 2001).

Fluorescence *in situ* hybridisation (FISH) with centromere-specific probes for chromosomes 1, 8, 12, 16 and X were performed according to standard protocols (Dracopoli, 2001). To analyse the copy number of 1p FISH with probes D1Z1 (1q12) and D1Z2 (1p36) was performed on 58 cases as described previously (Ambros *et al*, 1995; Hattinger *et al*, 1999, 2000). In 21 cases, DNA copy number changes were also determined by comparative genomic hybridisation (CGH) according to protocols described in detail previously (Armengol *et al*, 1997; Tarkkanen *et al*, 1999). For tumour DNA extraction, only tumour tissues containing more than 50% of tumour cells were accepted. In 11 cases without cytogenetic information, 22q12 rearrangements were studied by FISH using cosmid probes flanking the EWS breakpoint region at 22q12 according to a protocol previously described (Desmaze *et al*, 1994). EWS fusion transcripts were detected by RT–PCR in 59 cases as described (Delattre *et al*, 1994). In 45 patients, tumours were analysed by one of the methods (cytogenetics: 16, CGH: 5, FISH: 24), in 50 patients by two methods (cytogenetics and CGH, FISH or RT–PCR: 38; RT–PCR and CGH or FISH: 12), in 37 by three methods (cytogenetics and FISH and CGH or RT–PCR: 36; cytogenetics and CGH and RT–PCR: 1) and in two patients by all four methods. For the final evaluation of genetic parameters analysed, data obtained by all techniques were considered. In cases of disagreement between the results obtained by different techniques (two cases for del1p), this parameter was scored as not evaluable.

### Definition of genetic parameters for statistical evaluation

Numerical aberrations were evaluated as ‘gain’ or ‘loss’ of whole chromosomes. For statistical analysis ‘gain’ was defined as ‘plus one or more copies in addition to the appropriate somy of the analysed chromosome’. Isochromosomes were evaluated as ‘gain’. In samples that were analysed only by FISH, the numbers of centromere-specific hybridisation signals per nucleus were evaluated for at least two different chromosomes and 100 to 200 nuclei per sample were counted. For touch preparations and cyto spin slides at least 5% of the counted nuclei had to contain the abnormality. For paraffin embedded samples at least 7% of the nuclei had to show three hybridisation signals to be evaluated as trisomic (Zielenska *et al*, 2001). Structural aberrations were scored as ‘present’ or ‘not present’.

### Statistical analysis

Chi-square analysis was used to evaluate associations between clinical and genetic parameters and to test the consistency of data submitted from the different centres. Extent of disease at diagnosis (localised *versus* disseminated), tumour site (axial *vs* peripheral), age (<15 years *vs* ⩾15 years), sex, gain of chromosomes 8 and 12, gain of 1q, deletion of 1p and loss of 16q were the parameters analysed. The probability of overall survival (OS) and event-free survival (EFS) were estimated according to the method of Kaplan–Meier (Kaplan and Meier, 1958). Duration of EFS was computed from the date of diagnosis of ET to the first occurrence of disease, defined as local or systemic relapse or death. For the analyses of OS only death was considered as an event. The clinical follow-up was collected up to 31st March 2000. Estimates of the 5-year-probability of OS and EFS were given together with their 95% confidence intervals according to Dorey–Korn (Dorey and Korn, 1987). In addition, a proportional hazard model according to Cox (1972) was fitted. A stepwise selection procedure was used to identify the most important predictors among the genetic parameters, whereas all clinical parameters were forced to be included in the model. A *P* value ⩽0.25, after adjustment for the effects of other variables, was required for inclusion and retention in the model. The relative risk of failure (RHR) and the associated 95% confidence intervals were calculated with the coefficient and standard error from the Cox analyses. *P* values were from the likelihood ratio test. Additionally, alternative models were compared on the basis of Akaike's (1972) information criterion. All analyses were carried out for the whole population as well as for the group of patients with localised ET.

## RESULTS

### Genetic aberrations in Ewing tumours

Information on 22q12 rearrangements was available for 109 cases. The classical t(11;22)(q24;12) and/or an *EWS/FLI1* fusion transcript detected by classical cytogenetic analysis and/or RT–PCR, respectively, were found in 86 ETs (79%). A further 11 (10%) were positive for an *EWS* rearrangement by FISH using probes flanking the *EWS* breakpoint region on chromosome 22. Variant 22q12 rearrangements or deletions at 22q12 were found in 10 cases (9%). Two cases were highly positive for CD99 only.

The most frequent numerical aberrations, irrespective of subgroup, were gains of chromosomes 8 and 12, present in 68 (52%) and 36 (27%) of evaluable cases, respectively. Non-random structural aberrations additional to 22q12 rearrangements, involved the long arms of chromosomes 1 and 16 in 26 (21%) and 25 (21%) cases, respectively. In 14 of these cases (56%), gain of 1q and loss of 16q were the result of the unbalanced translocation t(1;16)(q10∼21;q10∼q13). Loss of whole chromosome 16 occurred in seven cases (28%). Deletions of the short arm of chromosome 1, with breakpoints ranging between 1p13-1p36.3, were found in 10 of the cases analysed (8%). Other non-random aberrations found in cases analysed by classical cytogenetic and/or CGH analysis were gain of chromosomes 20 (12 cases, 13%), 5 (12 cases, 12%), 2 (11 cases, 11%), 7 (10 cases, 10%), and 14 (nine cases, 9%). Isochromosomes of 8q were present in two cases, and in one case i(14)(q10) was found.

**Figure 1:**
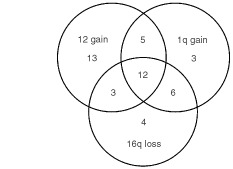
Distribution and interrelation of chromosome 12 gain, 1q gain and 16q loss in 46 out of 118 patients with Ewing tumours. None of these aberrations was found in Ewing tumours of 72 patients.

Frequencies and interrelations of chromosome 12 gain, 1q gain, and 16q loss in 118 ETs evaluated for all three parameters are shown in Figure 1

. The associations between these three genetic aberrations were statistically significant (*P*<0.0001). Interestingly, gain of chromosome 8 was only associated with gain of chromosome 12 (*P*<0.004). Seventy-two per cent of tumours with gain of chromosome 8 had also gain of chromosome 12 whereas only 44% of tumours with normal copy numbers of chromosome 8 had gain of chromosome 12. These associations were independent of whether the tumours were localised or disseminated. No association was found between 1p deletions and the other genetic parameters. The consistency of genetic data among the four countries was assessed by Chi-square analysis and no significant differences were detected (*P*>0.1 for all analyses).

### Correlations between genetic aberrations and clinical parameters

Associations between clinical parameters and genetic aberrations were found for 1q gain and 16q loss. Both aberrations correlated with age at diagnosis ⩾15 years (34% *vs* 13%, *P*=0.005 and 31% *vs* 15%, *P*=0.035). In the subgroup of patients with localised disease only 16q loss was still significantly associated with age ⩾15 years (*P*=0.047). The presence of 16q loss was also significantly associated with disseminated disease at diagnosis (32% *vs* 15%, *P*=0.038). Consistency among the four countries was seen for sex and tumour site (*P*>0.1), but not for age at diagnosis (*P*<0.0002). In Helsinki, more patients with an age older than 15 years at diagnosis were submitted compared to the other three centres.

### Analysis of overall and event-free survival

The median duration of follow-up for surviving patients was 5 years (range 8 months – 15 years). Five-year OS and EFS rates for all ET patients included for statistical analysis and for the subgroup of patients with localised ET at diagnosis are summarised in Tables 1

**Table 1:**
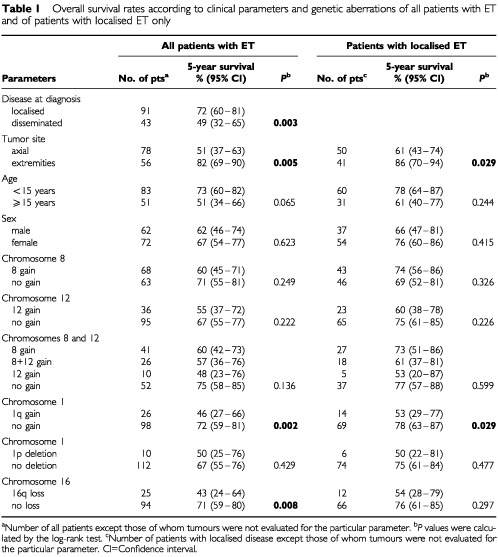
Overall survival rates according to clinical parameters and genetic aberrations of all patients with ET and of patients with localised ET only

and 2

**Table 2:**
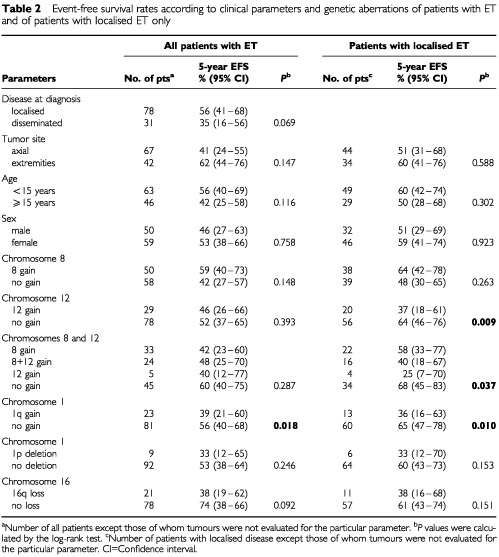
Event-free survival rates according to clinical parameters and genetic aberrations of patients with ET and of patients with localised ET only

. Patients with localised disease at diagnosis showed better OS than patients with disseminated disease (*P*=0.003). Univariate analysis of OS and EFS estimates revealed that tumour site was a predictive factor for OS as patients with axial tumours had poorer outcomes (*P*=0.005 and *P*=0.029).

**Figure 2:**
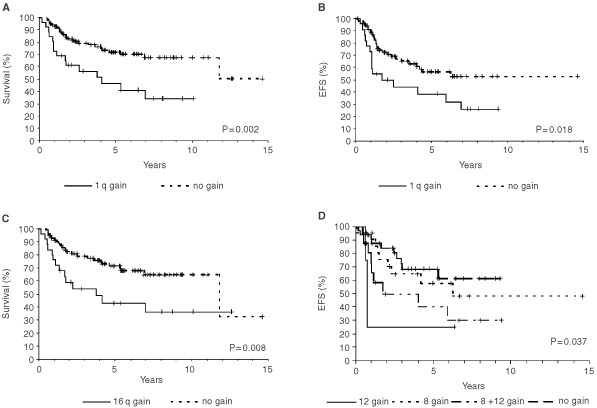
Overall survival plots (**A** and **C**) and event-free survival plots (EFS) (**B** and **D**) for patients with Ewing tumours displaying (**A**) 1q gain (*n*=26) or balanced ratios between 1p and 1q (*n*=98), (**B**) 1q gain (*n*=23) or balanced ratios between 1p and 1q (*n*=81), (**C**) 16q loss (*n*=25) or normal copy number of chromosome 16 (*n*=94), and (**D**) gain of chromosome 8 (*n*=22), gain of chromosomes 8 and 12 (*n*=16), gain of chromosome 12 (*n*=4) or normal copy numbers of chromosomes 8 and 12 (*n*=34).

Among the five genetic parameters tested, only gain of 1q was a predictive factor for both adverse OS and EFS either in the total group of patients (Figure 2A

,B and Tables 1 and 2) as well as in the subgroup of patients with localised disease (Table 2). Loss of 16q was significantly associated with adverse OS (*P*=0.008) (Figure 2C) but not with EFS in all patients, and did not show significant associations with outcome in the subgroup of patients with localised disease. In contrast, gain of chromosome 8 was not associated with clinical outcome in all evaluations. However, in patients with localised disease, gain of chromosome 12 was significantly associated with poorer EFS (*P*=0.009) and combined analysis of gain of chromosomes 8 and/or 12 identified four groups of patients with localised disease and statistically significant different EFS rates (Figure 2D).

In the multivariate model, gain of 1q maintained its significant impact on survival (RHR=2, *P*=0.046) after adjustment for clinical parameters. This was also true for loss of 16q (RHR=1.9, *P*=0.060). In a stepwise selection procedure only gain of 1q met the 25% level for entry into the model, which was expected because of the high correlation seen between gain of 1q and loss of 16q.

## DISCUSSION

In the present study, genetic and clinical data from 134 patients with skeletal and extraskeletal ETs were collected from four centres in order to determine whether chromosomal aberrations present in addition to 22q12 rearrangements are of prognostic significance. Diagnosis of ET was confirmed by high expression of the CD99 antigen and/or the presence of a 22q12 rearrangement. Tumour samples were analysed using a combination of classical cytogenetic analysis, FISH, CGH and/or RT–PCR.

In this series, evidence for 22q12 rearrangements was present in 98% of the cases analysed. Notably, as only 13% (11 out of 85) of the cytogenetically analysed cases did not display chromosomal aberrations in addition to a 22q12 rearrangement, this illustrates the importance of genetic analysis by complementary techniques to detect these additional genetic events. When compiling the data of all samples analysed, gain of chromosome 8 was found in 52% and of chromosome 12 in 28% of the cases. Gains of chromosomes 20, 5, 2, 7 and 14 were found in decreasing frequency in less than 14% of the cases and thus the results of the present study are consistent with previous studies (Kullendorff *et al*, 1999). The most frequent facultative structural aberrations were gains of 1q and loss of 16q that were present in 21% each. The majority displayed one or two der(16)t(1;16) chromosomes, thus having a genetic net imbalance of 1q gain and 16q loss, which was a statistically significant association. In addition, chromosome 12 gain was associated with gain of 1q and loss of 16q, but there was a relatively low frequency of ETs that displayed 1q gain and/or 16q loss that did not have gain of chromosome 12. These data suggest that tumours with gain of chromosome 12 might have a tendency towards acquiring structural alterations involving 1q and 16q and that these structural chromosomal aberrations occur later than numerical aberrations. Interestingly, although gain of chromosome 8 was the most frequent numerical aberration, it was only significantly associated with gain of chromosome 12 in this study. Deletions at 1p were not associated with one of the other frequent aberrations, although this is possibly due to deletions at 1p being present in only 8% of the cases analysed.

Previous studies have shown that the presence of metastases at onset is the main adverse prognostic parameter for patients with ET (Jürgens *et al*, 1988; Terrier *et al*, 1996; Cotterill *et al*, 2000). In the present study, disseminated disease at diagnosis and axial tumour site were significantly associated with adverse OS, although not with EFS. Among the genetic parameters evaluated for prognostic clinical impact gain of 1q was a strong predictor for adverse OS and EFS both in patients with localised disease and in the entire study group. Gain of 1q has also been reported to be associated with advanced disease stage in two other childhood tumours, neuroblastoma (Hirai *et al*, 1999) and Wilms' tumour (Hing *et al*, 2001). 1q is known to contain several genes that might contribute to the development and/or progression of human sarcoma (Forus *et al*, 1998; Knuutila *et al*, 1998). Loss of 16q correlated significantly with adverse OS in the entire study group but not in the subgroup of patients with localised disease. This observation could be due to the fact that 16q loss was significantly associated with disseminated disease at diagnosis. However, the biological consequences of 1q gain and 16q loss remain to be elucidated. Gains of chromosomes 8 and 12 for which trends toward worse outcome have been reported (Armengol *et al*, 1997; Tarkkanen *et al*, 1999) were not predictive factors for OS and EFS in the entire study group. In contrast, within the group of patients with localised ET gain of chromosome 12 identified a subgroup of patients with markedly poor clinical outcome.

Molecular studies have suggested that *TP53* alterations (de Alava *et al*, 2000), *INK4A* deletions (Wei *et al*, 2000), and type of the *EWS/FLI1* fusion transcript (Zoubek *et al*, 1994, 1996; de Alava *et al*, 1998) are predictive prognostic factors in ETs. Unfortunately, no study currently exists that provides molecular, cytogenetic, histopathological and clinical data of a series of patients large enough for statistical evaluation. The present study has shown that data from a series of 134 ET patients compiled from four different countries and obtained by complementary techniques enabled the identification of 1q gain as a negative prognostic marker for patients with ET regardless of stage of disease. In addition, loss of 16q is the first genetic parameter that is associated significantly with disseminated disease at onset. Therefore, the copy number of 1q and 16q should be evaluated in addition to the presence of a 22q12 rearrangement in ETs to identify patients with a higher probability of adverse outcome.
